# Alveolar ridge regeneration of damaged extraction sockets using deproteinized porcine versus bovine bone minerals: A randomized clinical trial

**DOI:** 10.1111/cid.12628

**Published:** 2018-07-27

**Authors:** Jung‐Seok Lee, Jae‐Kook Cha, Chang‐Sung Kim

**Affiliations:** ^1^ Department of Periodontology Research Institute for Periodontal Regeneration, Yonsei University College of Dentistry Seoul Republic of Korea; ^2^ Department of Applied Life Science, BK21 PLUS Project College of Dentistry Seoul Republic of Korea; ^3^ Department of Mechanical Engineering, College of Engineering Yonsei University Seoul Republic of Korea

**Keywords:** alveolar ridge reconstruction, bone grafting, extraction socket, randomized controlled trial, tooth extraction

## Abstract

**Backgrounds:**

Clinical benefits in bone grafting of intact extraction socket have been widely known, but limited evidence is available for the procedure in damaged extraction sockets due to periodontal disease.

**Purpose:**

This study aimed to determine the dimensional alteration of alveolar ridge following bone grafting of damaged extraction sockets, and compare the outcomes of using deproteinized bovine (DBBM) versus porcine bone mineral (DPBM) in the damaged sockets.

**Materials and Methods:**

One hundred patients (*n* = 50 for each group) with periodontitis‐induced damaged extraction socket were included in this randomized, single‐blind trial. After removal of tooth and granulation tissue, sites were grafted with either DBBM (DBBM group) or DPBM (DPBM group), and covered with collagen membrane. Linear/volumetric analyses of hard and soft‐tissue dimensions were performed on reconstructed/superimposed computed tomography and scanned cast images, taken immediately and 4 months after surgery.

**Results:**

The two groups showed comparable hard tissue augmentation with minimal reductions in the grafted volume, as well as in vertical (1.22 ± 2.16 and 1.45 ± 1.92 mm for DPBM and DBBM group, respectively) and horizontal (1.43 ± 3.40 and 1.83 ± 2.85 mm on the central section, respectively) dimensions at 4 months after surgery. However, several cases showed large variations in maintenance of the grafted volume. None of the measured parameters in hard and soft tissue dimensions differed significantly between DBBM and DPBM sites.

**Conclusions:**

DBBM and DPBM can comparably augment damaged extraction sockets with minimal postoperative reduction of the grafted volume. However, the large variations in the results should be further evaluated for application in routine dental clinics.

## 1 INTRODUCTION

The clinical procedure of grafting bone substitutes in the extraction tooth socket or peri‐implant circumferential defect around an immediately placed implant is based on preclinical studies that have shown dimensional shrinkage of the alveolar ridge after tooth extraction and the dimensional compensation achieved by placing slowly resorbed biomaterials in the extraction sockets.^1^, ^2^ Clinical studies have also demonstrated maintenance of the horizontal width of the alveolar ridge and favorable esthetic results achieved by bone grafting of extraction sockets and immediate implant placement with filling of bone gaps.^3^, ^4^ However, the preclinical and clinical studies supporting these techniques were all based on experimental models or clinical indications of a single tooth gap and an intact extraction socket in the anterior region.

The above‐mentioned indications are uncommon in the clinical setting, with a damaged extraction socket (missing one or more bony walls) occurring frequently due to periodontal and endodontic problems. This situation has prompted studies into extending the indications of extraction‐socket grafting,^5^, ^6^ as well as the use of biomaterials in these techniques.^7^, ^8^ Barone et al. demonstrated that the horizontal dimension of the alveolar ridge could be maintained after grafting biomaterials with coverage by a collagen membrane in buccal‐bone‐deficient extraction sockets in a single cohort study.^5^ Lee et al. also showed dimensional augmentation radiographically and successful new bone formation histologically by grafting bone substitutes and coverage with a collagen membrane in damaged extraction sockets in a preclinical animal model.^6^ These findings support the grafting of biomaterials even in cases of damaged extraction sockets with a deficient bony wall, which can be caused by periodontitis or a combined endodontic and periodontal lesion.

Most previous studies of ridge preservation techniques showed successful dimensional preservation using deproteinized bovine bone mineral (DBBM), whereas the use of other grafting materials having characteristics of rapid resorbability (eg, autogenous bone) resulted in relatively poor clinical outcomes in terms of dimensional alterations.^9^, ^10^ DBBM has, therefore, been a gold‐standard biomaterial for managing extraction sockets over the past 10 years based on the findings of both clinical and preclinical studies. However, recent clinical and preclinical studies have evaluated other types of grafting biomaterials as candidates for ridge preservation in order to minimize dimensional alterations of the alveolar ridge, and they have demonstrated comparably successful results both radiographically and histologically.^11^ These biomaterials can provide space maintenance that facilitates bone formation even when the thin buccal bone plate is resorbed, and can also compensate for dimensional alterations.^1^, ^12^

Xenografts from swine and equine origin were also introduced in the field of implant dentistry^13^, ^14^ due to a potential risk of bovine‐specific disease transmission by prions.^15^ Among these, xenografts from porcine were used in the clinical trials and animal studies based on structural similarities to human bone, with the report of successful bone formation and volume maintenance comparable to DBBM.^16^

The aims of this study were (i) to characterize dimensional alterations in alveolar ridge following grafting deproteinized bone mineral combined with coverage by a collagen membrane in damaged extraction sockets, and (ii) to compare the outcomes of using DBBM versus deproteinized porcine bone mineral (DPBM) in damaged extraction sockets in a randomized clinical trial.

## 2 MATERIALS AND METHODS

### 2.1 Study design and population

This study was designed as a randomized, single‐blinded clinical trial to compare DBBM (DBBM group; 0.25‐1.0mm, Bio‐Oss, Geistlich Pharma, Wolhusen, Switzerland) and DPBM (DPBM group; 0.25‐1.0 mm, THE Graft, Purgo Biologics, Seoul, Korea) in bone grafting of damaged extraction sockets. The experimental protocols were designed in accordance with the Helsinki Declaration (Tokyo version revised in 2004) and Good Clinical Practice Guidelines, and were approved by the Institutional Review Board for Clinical Research at the Dental Hospital of Yonsei University (Approval no. 2–2015‐0009).

Patients who were planned to receive tooth extraction due to severe periodontitis were included in the present trial. The patients were required to be at least 20 years old, able to understand the protocols, and provide voluntary informed consent to participate for this study. Patients with the following characteristics were excluded: smoking more than 10 cigarettes daily, uncontrolled diabetes, hypertension, or coagulopathy, osteomyelitis, any history of alcoholism, taking steroids or immunosuppressors within the previous 2 weeks, allergic reactions to porcine bone or bovine bone, any history of radiotherapy, or taking bisphosphonate medication.

### 2.2 Sample‐size determination

The required sample size was calculated using G*Power 3.1 software^17^ for a comparison of two experimental groups with a one‐sided alpha level of 5% and a statistical power of 90%. Based on previous results for the mean and standard deviation of horizontal changes in the ridge dimensions in two experimental groups,^18^ the effect size was calculated as 0.74. About 40 sites per group were required in the present study, and so 50 sites per group were used on the assumption of a dropout rate of 25%.

### 2.3 Randomization and blinding

Informed consents were obtained from all included participants after providing a detailed explanation of the protocols and the benefits of risks related to participation. Each participant was given an enrollment number and then randomly assigned to the DBBM or DPBM group using a sequentially numbered sealed‐envelope protocol. In brief, the participants were given enrollment‐number‐matched sealed envelopes containing group assignments based on block randomization.

At the next visit for experimental surgery, each participant was allocated immediately before applying the biomaterials after tooth extraction, by opening a sealed envelope. This trial was designed using single blinding, with all researchers blinded before opening the envelope, but then unblinded thereafter. Participants were blinded to their allocation. However, researchers were reblinded when performing measurements and analysis: a clinical research coordinator marked a concealed number on the radiographic and histologic data rather than the patient's information prior to performing the measurements.

### 2.4 Surgical protocols and timetable

**Figure 1:**
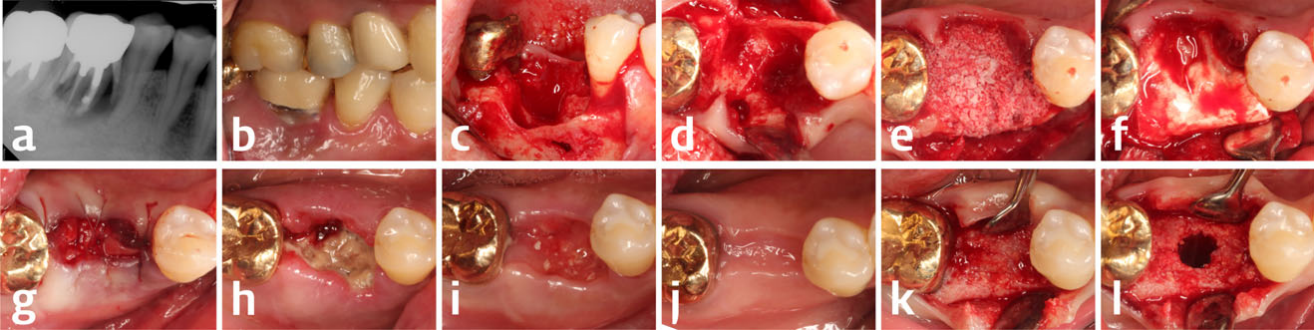
Clinical and radiographic photographs of the representative case in the present clinical trial (DPBM group). Initial periapical radiograph A, showed severe periradicular radiolucency related to vertical root fracture, and preoperative photograph B, revealed gingival inflammation around the tooth. After mucoperiosteal flap elevation and tooth removal (C and D), bony destruction extending to buccal/lingual bony walls and an interradicular septum could be found. Test bone substitute was grafted up to the extension line between the adjacent bony walls (E), and collagen membrane covered the graft (F). Delayed healing at the entrance of extraction socket was observed in the serial photographs; G, immediately, H, 1 week, I, 1 month, J, 4 months after the graft surgery. Regenerated alveolar ridge could be seen at the time of implantation with relatively poor density of bone (K and L)

Preoperative treatment for inflammation and infection control was performed at the first visit for screening, which involved subgingival scaling/root planning and the application of local antibiotics (minocycline hydrochloride; Periocline, Sunstar, Osaka, Japan) to the apical end of the lesion. These were also applied to other sites involved with periodontitis to subside pathologic signs and symptoms. Broad‐spectrum antibiotics were administered for 5–7 days before the extraction. On the second visit, a dental impression was taken for fabricating a cast, and the tooth was extracted gently under local anesthesia. Thorough curettage of granulation tissue was performed in the extraction socket so as to expose the bony surface in all regions of the socket. An incision was made from the adjacent dental papilla to an adjacent tooth to elevate a mucoperiosteal flap over the margin of the lesion, and the flap was elevated. After the exposed bony surface was clearly confirmed by performing further curettage, the DPBM and DBBM were grafted according to the random group allocation to fill the extraction socket so that the contour of the ridge was consistent with the adjacent ridge shape; the biomaterials were grafted up to the level at the extended outlines between the most coronal region of mesial and distal bony wall (vertically) and between the outermost regions of the adjacent alveolar ridge (horizontally). Collagen membrane (Bio‐Gide, Geistlich Pharma) was applied to cover the defect margin and the grafted biomaterials, and the mucoperiosteal flaps were repositioned and fixed with a 6‐0 nylon suture (Monosyn, B. Braun, Aesculap, Center Valley, PA, USA). Primary closure was achieved throughout the incised region except over the entrance of the extraction socket, which exposed the underlying collagen membrane but was also sutured to ensure wound stability. Gentle pressure was applied buccolingually on the grafted area for 3–5 minutes, and postoperative computed tomography (CT) was taken. Broad‐spectrum antibiotics were administered for 7 days. The suture was removed after a healing period of 1 week, and checkup visits were performed at 1 and 3 months after surgery (Figure 1A‐J).

CT and a dental impression were taken, and dental implants were placed 4 months after surgery. The bone was prepared for the implant using a trephine bur, and a tissue sample within the trephine bur was analyzed histologically (the histologic results are reported elsewhere).

### 2.5 Outcome variables

#### 2.5.1 Linear measurements

The alveolar ridge dimension was analyzed quantitatively on the CT images obtained immediately and 4 months after surgery (designated as the first and second CT images, respectively), and these images were automatically superimposed using computer software (OnDemand 3D, CyberMed, Seoul, Korea). The horizontal width and vertical height were measured as the primary outcomes in the same cross‐sectional plane as the superimposed view. The acquired CT images were processed in DICOM format, and the first and second CT images were fused automatically based on neighboring anatomic structures such as teeth, the mandibular border, and the sinus floor. The resulting superimposed CT image was aligned with the long axis of the adjacent tooth, and the grafted area was marked based on differences in radiopacity relative to the preexisting neighboring bone (Figure 3A‐E). An imaginary bottom plane was drawn that included the lowermost point of the grafted area on the first CT image and was perpendicular to the previously set axis. The vertical height was measured as a distance from the bottom plane to the uppermost point of regenerated alveolar ridge on the superimposed CT image. The horizontal width was measured at three lines that divided the grafted area equally into four sections (Figure 4A and B). The changes in the vertical height and horizontal width were calculated by subtracting the measurements from the first and second CT images.

#### 2.5.2 Volumetric measurements

The secondary outcomes were changes in the grafted volume, papilla height, and soft‐tissue dimensions. The grafted volume within the damaged extraction socket was measured on the reconstructed CT images taken immediately after surgery. The volume change was also measured on the superimposed CT image. An imaginary cube was set that included the bottom plane and which contacted the most‐prominent contour of the grafted area, and the volume change was calculated on the superimposed CT image using computer software. The percentage volumetric change relative to the grafted volume was calculated.

#### 2.5.3 Papilla height and soft‐tissue dimensions

Papilla height was measured on the CT images, and horizontal/vertical soft tissue dimensions were measured on the superimposed images of two scanned‐cast and CT data. The papilla height was measured on cross‐sections of CT images from both the mesial and distal ends of the grafted area as the distance from the uppermost grafted/regenerated point to the uppermost soft‐tissue level on the gingival crest.

The soft‐tissue dimensions were measured using superimposed scanned data from the cast; dental impression taken immediately before and 4 months after surgery. The two superimposed scanned‐cast images and the first CT image were reoverlapped using the aforementioned method, with the resulting image positioned parallel to the long axis of the adjacent tooth. The vertical reduction of gingival margin was measured at the buccal and lingual surfaces on images from the center of the site, and the horizontal reduction of the soft‐tissue width was measured at the top of the grafted area (Figure 3F).

#### 2.5.4 Statistical analysis

The mean ± standard‐deviation values of all parameters in the radiographic and cast analyses were calculated for the DBBM and DPBM groups. Unpaired *t*‐tests were used to determine the significance of any differences between the two groups, with the cutoff for statistical significance set at *P* < .05.

## 3 RESULTS

### 3.1 Participants

**Figure 2:**
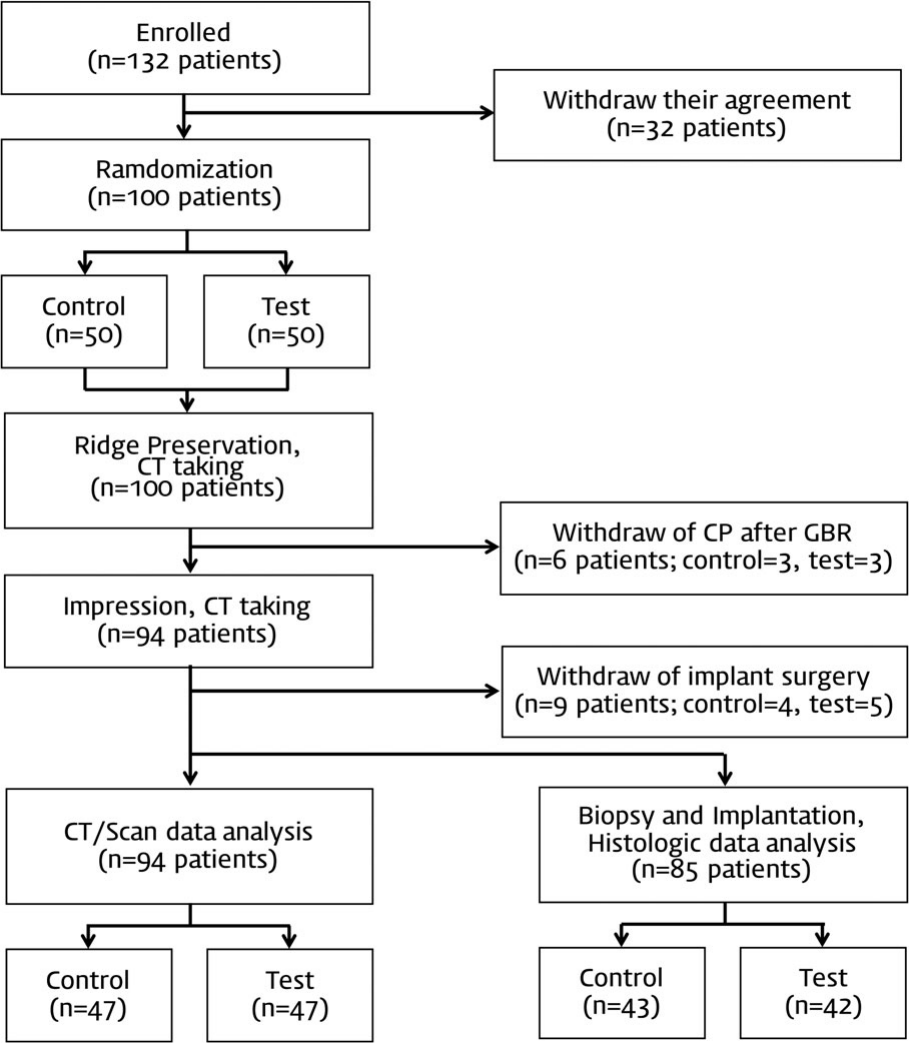
Flow chart illustrating the procedure of the clinical trial

In total, 132 volunteers were screened in this study from December 2015 to October 2016, with 100 patients finally included. In cases where multiple teeth or sites were extracted, the most severely damaged site was chosen for inclusion in this clinical trial. The 100 sites were randomly assigned to the DBBM (*n* = 50) and DPBM (*n* = 50) groups, and they received ridge regeneration with a collagen membrane and the applicable biomaterial. Six participants were lost during the follow‐up period before implant surgery, and nine participants withdrew their permission for implant installation; however, CT and cast impressions were still taken in the latter nine participants. Therefore, the procedures for this trial were ultimately completed in 94 patients, with 47 in each group included in the quantitative radiographic and cast analyses (Figure 2).

**Figure 3:**
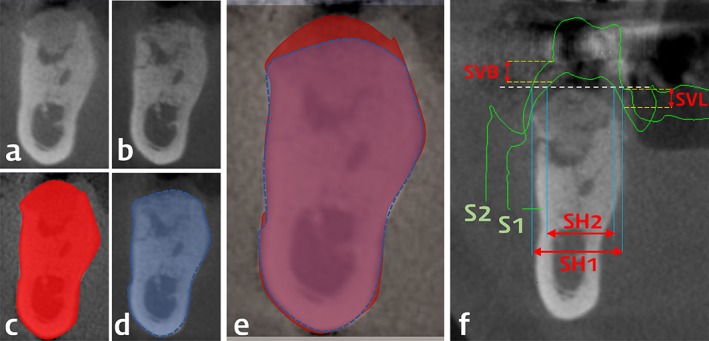
Representative radiographs and overlapped 3D‐scanned study casts (the same case with Figure 1) and linear measurements. Cross‐sectional view from computed tomography taken at the time of surgery (A and C) and 4 months after the surgery (B and D). In the automatically superimposed images from the two time‐points (E), vertical and horizontal changes of the alveolar bone dimension were measured. Surface information from the 3D‐scanned study casts (S1 at the time of surgery; S2 at 4 months after the surgery) were superimposed with the computed tomography at 4 months after the surgery (F). In this view, horizontal soft tissue dimension was measured at the most‐crestal level of the regenerated alveolar bone at two time‐points (SH1 and SH2), and horizontal reduction was calculated by their subtraction. Vertical change of soft tissue dimension was measured at both buccal and lingual gingival margins around the extracted tooth (SVB and SVL)

The demographic data of the finally included participants are presented in Table 1; none of their characteristics differed significantly between groups. Most of the sites included in this study were in the molar area, and the site distribution was similar in the two groups. In total, sockets involving the extraction of 79 single tooth sites and 15 multiple teeth sites were included, with no significant difference in the proportion between the two groups.

**Table 1:** Demographic results of the participants in this study

	DBBM group (*n* = 47)	DPBM group (*n* = 47)
**Age (years; mean ± SD)**	54.47 ± 11.00	56.13 ± 10.20
**Gender (male/female)**	32/15	32/15
**Site (incisor/premolar/molar)**	5/6/36	2/8/37
**No. of extracted teeth (single/multiple)**	40/7	39/8
**Smoking (nonsmoker/smoker)**	44/3	42/5

There was no significant difference in distribution of all samples between control and test groups.

DBBM, deproteinized bovine bone mineral; DPBM, deproteinized porcine bone mineral; Smoker, smoking less than 10 cigarettes daily.

### 3.2 Clinical findings

The included sites exhibited defects with various morphologies, including circumferential defects and the destruction of one or two bony walls. Some parts of gingival margin had receded in the exposed area of the extraction‐socket entrance, and sloughing tissues covered the entire expanded socket entrance at 1 week after the surgery. Patients reported experiencing moderate‐to‐severe pain for 2–5 days after surgery. Delayed healing in that area resulted in the transient loss of the dental papilla in most of the experimental sites (36 of 46 in the DBBM group and 35 of 44 in the DPBM group). However, complete coverage of soft tissues was seen in that area at 1 month after the surgery. Grafted particles were observed in this soft tissue, in which exposed collagen membrane might be resorbed. Regenerated alveolar ridge was evident 4 months later, and dental implants could be installed and restored by fixed prostheses successfully at all of the included sites (Figure 1K and L). Additional grafting procedure such as guided bone regeneration was performed in 13 sites in DBBM group and 8 sites in DPBM group, showing insufficient bone volume for implantation owing to dimensional shrinkage.

### 3.3 Linear measurements

**Figure 4:**
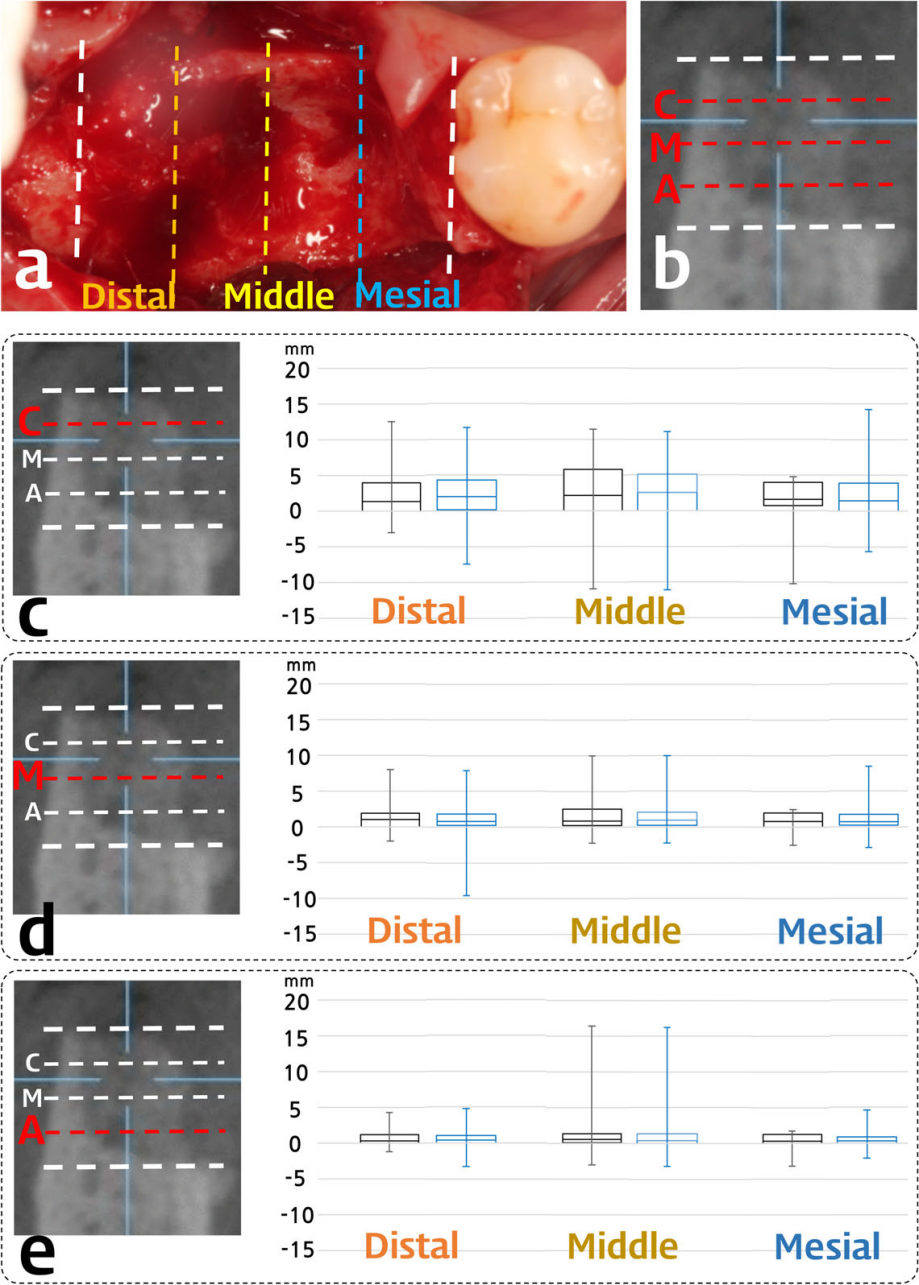
Box plots of the horizontal width reduction of the DBBM (black) and the DPBM group (blue). Box plot revealed the first and the third quartiles, median, and maximum/minimum of the data. C, coronal; M, middle; A, apical level

**Figure 5:**
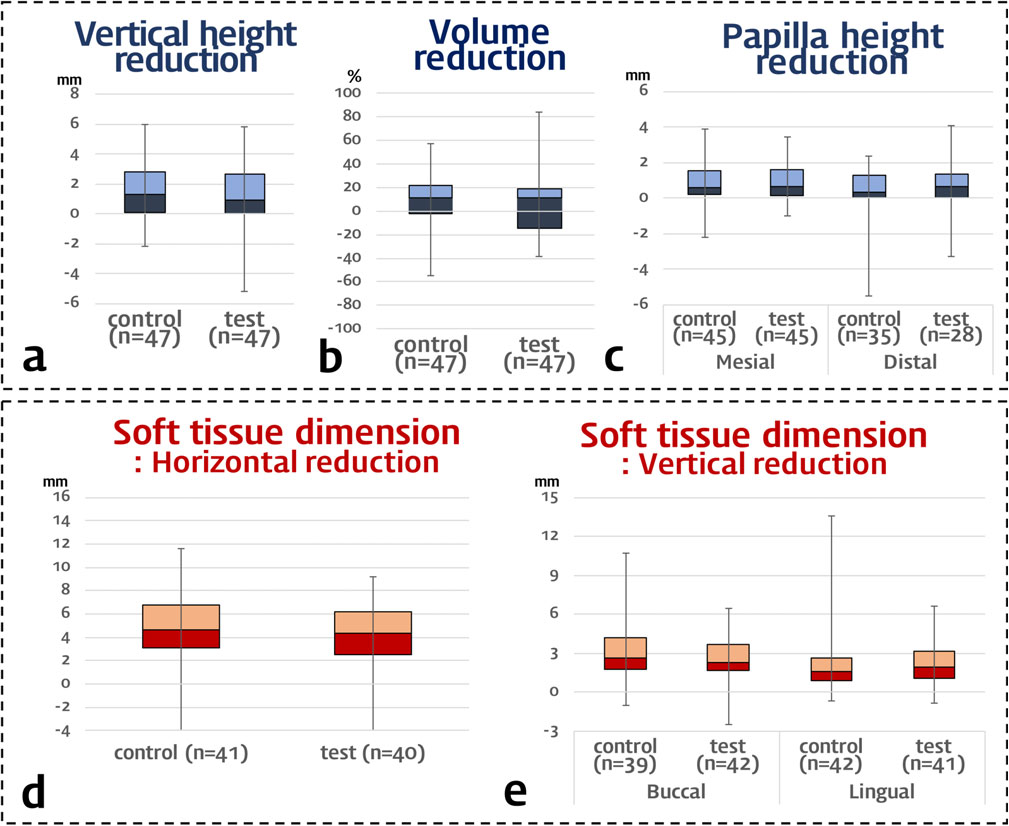
Box plots of the vertical height and volume reduction in alveolar ridge and changes in soft tissue dimension. Box plot revealed the first and the third quartiles, median, and maximum/minimum of the data

The radiographic alterations of the horizontal and vertical dimensions are presented in Table 2, Figures 4 and 5. The overall grafted ridge width was reduced by less than 2 mm (1.43 ± 3.40 and 1.83 ± 2.85 mm for DPBM and DBBM group, respectively) on the central section of the grafted area, but there were no differences between the DPBM and DBBM groups. The horizontal width was minimally altered at both the apical and middle levels; 0.62 ± 1.66 and 1.16 ± 2.15 mm for DPBM group, 0.73 ± 1.40 and 1.22 ± 1.89 mm for DBBM group, respectively. At the coronal level, the horizontal dimension had reduced by more than 2 mm (2.09 ± 4.19 and 2.67 ± 3.42 mm for DPBM and DBBM group, respectively) across all measured sections (distal, middle, and mesial), and wider variation was observed compared with lower levels (apical and middle). In addition, there was a tendency for the range of horizontal width alterations to be larger in the middle than the distal and mesial sections of the grafted socket area. Both DBBM and DPBM groups showed similar reductions of the horizontal width at all levels and similar range variations in all sections (Figure 4C‐E).

**Table 2:** Results from linear/volumetric measurements on computed tomography and 3D‐scanned data of study casts

Horizontal width reduction (mm)			DBBM group (*n* = 47)			DPBM group (*n* = 47)		
			Apical	Middle	Coronal	Apical	Middle	Coronal
	**Middle**		1.21 ± 2.69	1.59 ± 2.34	2.81 ± 4.47	0.84 ± 2.08	1.43 ± 1.77	2.50 ± 4.73
	**Distal**		0.74 ± 1.32	1.08 ± 1.86	2.33 ± 3.35	0.60 ± 1.34	1.47 ± 1.85	2.24 ± 3.52
	**Mesial**		0.66 ± 1.51	1.01 ± 1.32	2.47 ± 3.05	0.59 ± 1.34	1.1 ± 1.68	1.97 ± 3.95
	**Average**		0.73 ± 1.40	1.22 ± 1.89	2.67 ± 3.42	0.62 ± 1.66	1.16 ± 2.15	2.09 ± 4.19
**Vertical height reduction (mm)**			1.45 ± 1.92			1.22 ± 2.16		
**Volume reduction (%)**			8.14 ± 22.23			6.48 ± 24.23		
**Papilla height reduction (mm)**	**Mesial**		0.82 ± 1.22			0.87 ± 1.21		
	**Distal**		0.15 ± 1.60			0.23 ± 1.65		
**Soft tissue dimension (mm)**	**Horizontal reduction**		4.74 ± 3.14			4.04 ± 2.92		
	**Vertical reduction**	**Buccal**	3.00 ± 2.06			2.63 ± 1.90		
		**Lingual**	2.08 ± 2.23			2.17 ± 1.67		

A, apical; M, middle; C, coronal. There was no significant difference in all parameters between control and test groups.

The augmented height was reduced vertically by 1.45 ± 1.92 mm in the DBBM group and 1.22 ± 2.16 mm in the DPBM group. The magnitude of the reduction was similar, along with similar variations in range, with the exception of a single case in the DPBM group that exhibited increase in vertical height of 5.20 mm (Figure 5A).

### 3.4 Volumetric measurements

There were similarly small volumetric changes in the DBBM and DPBM groups (Table 2); the proportional volumetric changes relative to the grafted volumes were 8.14% ± 22.23% and 6.48% ± 24.23% in the DBBM and DPBM groups, with a very large variation in range [from minimum −38.90 (−54.80) to maximum 40.21 (57.65)% for DPBM (DBBM) group] (Figure 5B).

### 3.5 Papilla height and soft‐tissue dimensions

The papilla height was not measured in some parts of samples showing overlap of the papilla and other soft tissues such as buccal cheek in CT images: the mesial papilla height was measured in 45 of the 47 samples in each DBBM and DPBM group, while the distal papilla height was measured in 35 of the 47 samples in the DBBM group and in 28 of the 47 samples in the DPBM group. The results for the reduced number of samples showed reductions of the papilla height of less than 1 mm, but with greater reductions in mesial rather than distal papilla heights in both the DBBM and DPBM groups: 0.82 ± 1.22 mm in the DBBM group and 0.87 ± 1.21 mm in the DPBM group for the mesial papilla height, and 0.15 ± 1.60 mm in the DBBM group and 0.23 ± 1.65 mm in the DPBM group for the distal papilla height (Figure 5C).

Several samples were also excluded when measuring the soft‐tissue dimensions on cast images due to a lack of information about the measuring points on the casts due to severe recession over the oral vestibule. The number of included samples and measured data (horizontal alterations of soft tissue and vertical alterations of buccal or lingual soft tissues) in the cast analyses are presented in Figure 5D and E. Despite the exclusion of these samples, the results revealed similar mean and median values with similar ranges for all measurements of the horizontal and vertical soft‐tissue dimensions.

## 4 DISCUSSION

The present study performed the grafting two types of biomaterials (DBBM and DPBM) in damaged extraction socket, which included periodontitis‐induced circumferential defects and/or the destruction of one/two bony walls. Minimal dimensional alteration within 2 mm were shown in both horizontal and vertical aspects, but with large variations. These were in agreement with a recent multicenter clinical study of grafting at buccal‐bone‐deficient extraction sockets^18^ showing significantly larger dimensional alterations and a much larger standard deviation (2–4 mm) than previous results for intact socket grafting. Previous clinical studies^19^ of alveolar ridge preservation in intact extraction sockets have found minimal changes in the horizontal and vertical dimensions by grafting xenogenous and allogenous bone substitutes, with small ranges for each value (around 1 mm in most of these studies). Another single‐cohort clinical study^5^ of grafting at damaged extraction sockets found minimal changes in dimensions along with small variations, which was comparable to the results obtained for intact extraction sockets. While both of these previous studies were similarly based on indications for buccal‐bone‐deficient extraction sockets, the region of the experimental sites differed: the study of Barone et al. was limited to the anterior region, whereas that of Scheyer et al. included extraction sites in the posterior region.

A significantly larger defect and socket entrance in the molar region would affect the healing following grafting and dimensional shrinkage of the grafted at a damaged socket, which were also in accordance with another previous study^20^ showing an increased frequency of dehiscence defect formation for fixtures implanted in the grafted molar region. The present study also included molar extraction sites, and most of the experimental sites were in the posterior region. These features may result in differences in healing patterns in terms of dimensions, such as reductions in the maintenance of the horizontal width and vertical height along with larger variations.

Delayed soft tissue healing by large entrance of “damaged” extraction socket in “molar” region would have resulted in large variations in outcomes of this study. The previous study comparing flap and flapless ridge preservation technique showed significantly increased alteration in horizontal dimension, and this study also showed delayed healing in soft tissues extended to papilla region (Figure 1). However, papilla height was recovered with the minimally decreased level within 1 mm compared with the preexisting papilla height. Although there were several numbers of cases showing gingival recession of the adjacent teeth, these might not affect the variety of the dimensional alteration.

A recent clinical study^21^ that focused on indications for molar extraction sites found beneficial effects of extraction‐socket grafting in terms of maintaining the vertical dimension, but not the horizontal dimension. Although those authors excluded sites with periodontal destruction, the horizontal dimension at the coronal level was reduced by 2‐2.5 mm at 3 months compared with immediately after extraction/grafting. These findings are similar to the present results obtained in both the DBBM and DPBM groups, of 2.33‐2.81 and 1.97‐2.50 mm, respectively. In terms of maintaining the vertical height, extraction‐socket grafting in the previous study^21^ and the present study produced similar values. Considering the high proportion of posterior sites in the present results (36 of 47 and 37 of 47 in the DBBM and DPBM groups, respectively), the horizontal width and vertical height could be maintained by extraction‐socket grafting even at periodontally compromised sites to levels comparable to those for an intact extraction socket. Therefore, grafting immediately after extraction is expected to prevent dimensional collapse in a totally or partially damaged extraction socket, and also to reduce the need for additional surgery such as guided bone regeneration or sinus augmentation (Supporting Information Figure S1).^20^, ^22^

The technique of extraction‐socket grafting was originally developed in preclinical experiments using DBBM, but several recent clinical studies have used or compared various types of bone graft biomaterials and/or membranes. Previous studies involving conventional extraction‐socket grafting in intact sockets produced comparable results in terms of maintenance of the alveolar ridge dimension.^8^, ^11^, ^23^, ^24^, ^25^ However, in a recent study of damaged extraction sockets, two types of graft biomaterials (allogenous bone graft with a cross‐linked collagen membrane and DBBM with a non‐cross‐linked collagen membrane) produced significantly different patterns of dimensional alterations of the alveolar ridge.^18^ The present study compared two bone graft biomaterials using the same non‐cross‐linked collagen membrane, and obtained similar results for horizontal and vertical dimensional alterations along with similar ranges of values. Another previous clinical study comparing these two biomaterials in a sinus augmentation procedure found similar volumetric maintenance patterns and histologic new bone formation properties. In the present study, the similar healing patterns when using DBBM and DPBM resulted in similar dimensional changes in the alveolar ridge in cases with damaged extraction sockets.^26^

This study specifically included periodontitis‐involved lesion only, but this inclusion criterion poses itself as a limitation. Various types of defects were included, and these could have affected large variations of dimensional outcomes. Additional grafting procedures were performed in several cases showing extensive shrinkages in dimension (24.71%; 30.23% for control and 19.05% for test group), and yet, this requires further studies to confirm the clinical necessity of these procedures in damaged extraction sockets. In addition, the issues for appropriate protocol such as intentional membrane exposure via socket entrance and healing time for implant installation should be evaluated in further studies. Therefore, this study should be interpreted conservatively, as a pilot study for the extended indication of extraction socket grafting.

This study found minimal alterations in alveolar ridge volume both horizontally and vertically after grafting either type of deproteinized bone mineral in damaged extraction sockets induced by periodontitis‐involved lesion, which was comparable to the findings of previous investigations of intact extraction sockets. It is therefore suggested that DBBM and DPBM can be similarly used for grafting in damaged extraction sockets that lack bony walls. However, the presence of very wide ranges of values for all of the dimension‐related parameters means that further studies are needed to narrow the indications for damaged extraction sockets in order to enhance the predictability of extraction‐socket grafting techniques.

## CONFLICT OF INTEREST

The authors declare that they have no conflicts of interest with the contents of this article.

## Supporting information


**FIGURE S1** Radiographs and clinical photographs from a representative case showing a reduction of maxillary sinus pneumatization by extraction socket grafting (control group). Initial periapical radiograph (A) and postoperative serial radiographs (B, immediately after grafting; C, 4 months later; D, 1 year from extraction socket grafting). (E to H) intraoperative clinical photographs; (I) 1 week after grafting; (J to L) implant surgery at 4 months after grafting
