# Supplementary figures and images for: Alveolar ridge regeneration of damaged extraction sockets using deproteinized porcine versus bovine bone minerals: A randomized clinical trial
Source: Clin Implant Dent Relat Res. 2018 Jul 27;20(5):729–37. doi: 10.1111/cid.12628 (PMC6220803; doi:10.1111/cid.12628)

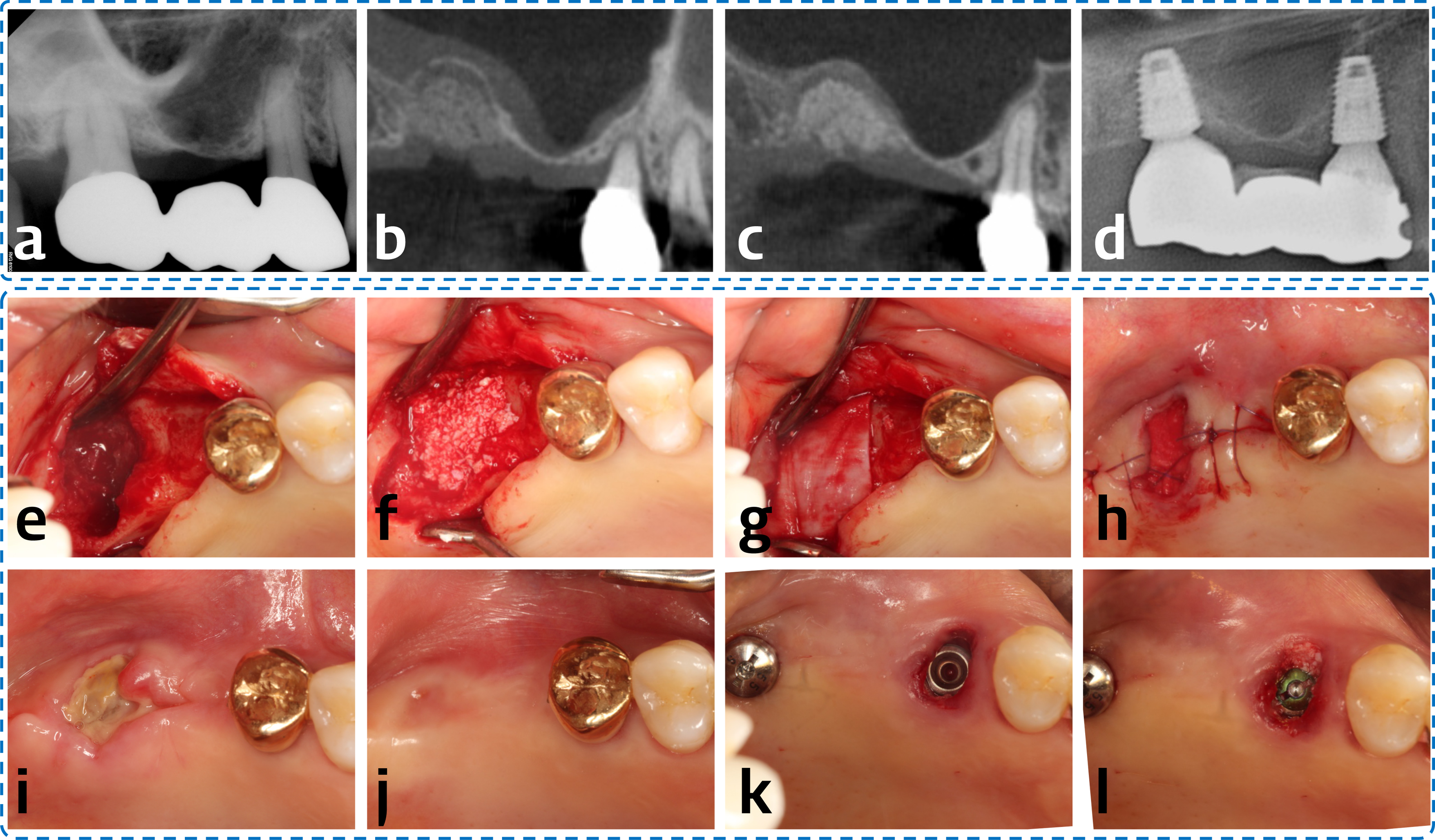

Supplement: Supplementary file 1 — FIGURE S1 Radiographs and clinical photographs from a representative case showing a reduction of maxillary sinus pneumatization by extraction socket grafting (control group). Initial periapical radiograph (A) and postoperative serial radiographs (B, immediately after grafting; C, 4 months later; D, 1 year from extraction socket grafting). (E to H) intraoperative clinical photographs; (I) 1 week after grafting; (J to L) implant surgery at 4 months after grafting [file CID-20-729-s001.tif]
